# Artificial intelligence-based approaches for advance care planning: a scoping review

**DOI:** 10.1186/s12904-025-01827-x

**Published:** 2025-10-23

**Authors:** Umut Arioz, Matthew John Allsop, William D. Goodman, Suzanne Timmons, Kseniya Simbirtseva, Izidor Mlakar, Grega Mocnik

**Affiliations:** 1 Faculty of Electrical Engineering and Computer Science, University of Maribor, Maribor, 2000 Slovenia; 2 School of Medicine, Faculty of Medicine and Health, Leeds Institute of Health Sciences, University of Leeds, Leeds, UK; 3 Centre for Gerontology and Rehabilitation, School of Medicine, College of Medicine and Health, University College Cork, Cork, Ireland

**Keywords:** Advance care planning, Digital tools, Palliative care, Artificial intelligence, Machine learning

## Abstract

**Background:**

Advance Care Planning (ACP) empowers individuals to make informed decisions about their future healthcare. However, barriers including time constraints and a lack of clarity on professional responsibilities for ACP hinder its implementation. The application of artificial intelligence (AI) could potentially optimise elements of ACP in practice by, for example, identifying patients for whom ACP may be relevant and aiding ACP-related decision-making. However, it is unclear how applications of AI for ACP are currently being used in the delivery of palliative care.

**Objectives:**

To explore the use of AI models for ACP, identifying key features that influence model performance, transparency of data used, source code availability, and generalizability.

**Methods:**

A scoping review was conducted using the Arksey and O’Malley framework and the PRISMA-ScR guidelines. Electronic databases (Scopus and Web of Science (WoS)) and seven preprint servers were searched to identify published research articles and conference papers in English, German and French for the last 10Â years’ records. Our search strategy was based on terms for ACP and artificial intelligence models (including machine learning). The GRADE approach was used to assess the quality of included studies.

**Results:**

Included studies (*N* = 41) predominantly used retrospective cohort designs and real-world electronic health record data. Most studies (*n* = 39) focused on identifying individuals who might benefit from ACP, while fewer studies addressed initiating ACP discussions (*n* = 10) or documenting and sharing ACP information (*n* = 8). Among AI and machine learning models, logistic regression was the most frequent analytical method (*n* = 15). Most models (*n* = 28) demonstrated good to very good performance. However, concerns remain regarding data and code availability, as many studies lacked transparency and reproducibility (*n* = 17 and *n* = 36, respectively).

**Conclusion:**

Most studies report models with promising results for predicting patient outcomes and supporting decision-making, but significant challenges remain, particularly regarding data and code availability. Future research should prioritize transparency and open-source code to facilitate rigorous evaluation. There is scope to explore novel AI-based approaches to ACP, including to support processes surrounding the review and updating of ACP information.

## Introduction

Advance Care Planning (ACP) “…is a voluntary process of person-centred discussion between an individual and their care providers about their preferences and priorities for their future care” [1]. This often includes multiple discussions and can include any people that a person would like to involve. The goal of ACP is to ensure that a person's values, beliefs, and goals for care are understood and respected, particularly when they may be unable to communicate these wishes themselves [2]. This process is especially important when individuals face serious illness, empowering them to articulate their healthcare preferences and appoint a surrogate decision-maker. ACP may involve documenting these preferences through advance directives or similar legally binding instruments to ensure patient-centered care, even in moments when they cannot advocate for themselves. ACP is not limited to palliative care settings but spans community and acute care environments [3]. It plays a central role in ensuring that healthcare aligns with patient values throughout their illness trajectory. Caregivers, in their supportive roles, can help ensure that these values are continually reassessed and that the patient's healthcare preferences evolve as needed. The regular review of ACP is essential to reflect changes in health status or personal values, further enhancing the quality of care at the end of life [4]. Moreover, early engagement in ACP, involving both the patient and their caregivers, has been shown to reduce unnecessary interventions, mitigate healthcare costs, and improve the overall quality of care by focusing on patient-centered outcomes, especially in populations such as cancer patients [5, 6]. Evidence highlights that when caregivers understand the patient's goals, they experience reductions in both anxiety and emotional strain as they feel more prepared to navigate complex medical decisions [7].

Despite the potential benefits of ACP, its implementation remains a challenge for healthcare professionals. Insufficient communication skills, a lack of knowledge to determine when and how to have ACP conversations, and time constraints hinder its widespread adoption [8]. While time constraints are a significant barrier, an emerging approach to support ACP implementation is the integration of Artificial Intelligence/Machine Learning (AI/ML) methods which offers broader potential benefits beyond simply saving time. Artificial Intelligence (AI) encompasses various methods that enable machines to mimic human cognitive functions. Among these, Machine Learning (ML) is a dominant and widely used subset, where systems learn from data to perform tasks without being explicitly programmed. In that sence, AI can contribute to ACP by, for example, supporting the timely identification of people who could benefit from ACP through algorithms that predict mortality and disease progression [4, 7, 9–14]. Furthermore, AI can enhance predictive accuracy in identifying individuals who would benefit most from ACP discussions [15–18] personalize ACP processes by analyzing patient data to tailor information and discussions to individual needs and values [19–22] and support clinicians with decision-making tools that integrate real-world data to forecast patient outcomes and facilitate more informed end-of-life care planning [1, 20, 23–25].

While digital resources such as websites, portals, and apps have been designed to support reflection, communication, decision-making, and documentation of end-of-life care, the specific application of artificial intelligence approaches in this context remains relatively understudied [26]. AI-powered tools differ from traditional digital platforms by offering enhanced interactivity, prediction, and data-driven personalization. For instance, AI can facilitate more interactive experiences through chatbots or conversational agents that guide users through ACP discussions [27]. Predictive AI models can analyze patient data to anticipate future care needs or identify individuals who might benefit most from ACP, enabling proactive engagement [28]. Furthermore, AI algorithms can analyze individual patient characteristics to tailor information and support, adapting to specific preferences and circumstances in a way that static websites or portals cannot [29]. While AI-based approaches are beginning to show promise in identifying patients in their last year of life, few resources integrate seamlessly with existing healthcare workflows or offer interactive interfaces tailored for healthcare providers [30]. For the specific use of AI-based approaches to support ACP, to date, there has been no compilation and outline of the current evidence base. This scoping review aims to comprehensively examine the current state of AI approaches within digital tools for ACP. Specifically, it explores how AI approaches can be applied to support ACP as part of palliative care delivery.

## Methods

### Overview

This scoping review was undertaken as part of the AI4HOPE project (https://www.ai4hope.eu). The review was undertaken as part of early co-design activities to guide the development of a digital ACP platform to support information provision for people living with mild to moderate dementia, alongside a focus on the documentation of information that they would like to be part of ACP discussions,. As the topic of AI supporting ACP is still emerging, this review focuses on any health conditions. The scoping review questions were formed through applying the Population-Concept-Context (PCC) framework [31]. ‘Population’ included people with serious or life-limiting illnesses, ‘Concept’ included the application of AI approaches as part of the ACP process, and ‘Context’ remained broad and included any country setting internationally, alongside including any care setting supporting the population of interest. Three broad questions derived from the PCC framework elements guided the approach to the scoping review, and included: In which domains of ACP can AI approaches be effectively applied? What are the primary and secondary objectives of utilizing AI approaches in digital tools for ACP? What are the most critical features to consider in AI approaches for ACP, and how can feature selection be optimized to enhance model performance and generalizability? This scoping review adhered to the methodological framework outlined by Arksey and O'Malley [32] and complies with the PRISMA-ScR checklist [33]. Following this guidance, we conducted six sequential steps: 1) defining research questions, 2) developing a comprehensive search strategy, 3) selecting studies based on predefined criteria, 4) extracting and analyzing data, 5) summarizing and reporting findings, and 6) consulting study authors for validation. The PRISMA-ScR checklist ensured the systematic, transparent, and comprehensive nature of our review.

### Eligibility criteria

The search strategy focused on people with serious or life-limiting illnesses, irrespective of setting. The emphasis on AI approaches supporting ACP highlights the importance of the palliative care setting but the review retains a broader focus across any care setting supporting the population of interest. Database searching included published research articles and conference papers between 2015 and 2024. Studies in three languages were included (English, German and French).

### Information sources

Scopus and Web of Science (WoS) databases were used to identify relevant literature. Also, the following preprints servers were searched: arXiv, bioRxiv, JMIR Preprints, medRxiv, Preprints.org, Research Square and SSRN Journal.

### Search

Our search strategy was based on the ‘AND’ operation of two main categories; keywords related to ACP, and keywords related to AI approaches. The box below provides examples of key terms used in the search strategy.

**Table:** 

("advance care planning"OR"advance care directives"OR"advance directives"OR"advance statement"OR"advance decision"OR"advanced care planning"OR"advanced care directives"OR"advanced directives"OR"advanced statement"OR"advanced decision"OR"palliative care"OR"Palliative Medicine") AND ("Artificial intelligence"OR"machine learning"OR"multivariate approach"OR"AI-enabled"OR"ML-generated"OR"Supervised learning"OR"unsupervised learning"OR"Deep learning"OR"classification"OR"Neural networks")	

### Selection of sources of evidence

A comprehensive literature search yielded a substantial number of potential studies. Prior to this independent screening, we conducted a calibration exercise. All authors participated in discussions to refine the eligibility criteria and assessed collectively a sample dataset comprising more than 10% of the total papers. We discussed our selections and the rationale behind them, which led to further refinement of the eligibility criteria. Following this calibration, the remaining papers were divided uniformly among the authors for independent title and abstract screening. In the second screening phase, all authors conducted a detailed review of the full texts of the remaining studies. The same exclusion criteria as in the previous stage were applied. Discrepancies were resolved through consensus among the authors.

### Data collection and charting process

Relevant data were extracted from the final list of studies and organized into a spreadsheet by all authors.

### Data items

The data extraction characteristics chosen for the scoping review provide an overview of key characteristics of included studies. Data categories extracted included Study Identification and Design (the target disease(s) or condition(s) being investigated; study design; and number of participants); Data and Methodology (origin of the data, such as EHRs or publicly available databases, and number of records in the dataset); Application and Evaluation of AI approaches (the specific algorithms employed in the study, with categories derived from existing literature [34–36]); primary and secondary aim(s); data types included in models (specific characteristics or variables extracted from the data for analysis); data availability (whether the study dataset is publicly accessible and, if so, from where, including the source code to support replication and further exploration); model performance (evaluation metrics used to assess the accuracy and effectiveness of the AI approach, such as sensitivity, specificity, or accuracy); and clinical application within ACP processes. For the clinical application of processes, included studies were charted against different elements that may be involved in the identification, documentation, revision and sharing of ACP documentation. The phases were informed by an earlier conceptual model of digital approaches to ACP [37]. Studies were classified against any of the six elements of ACP (Appendix D), outlined in Fig.Â 1.

**Fig.Â 1:**
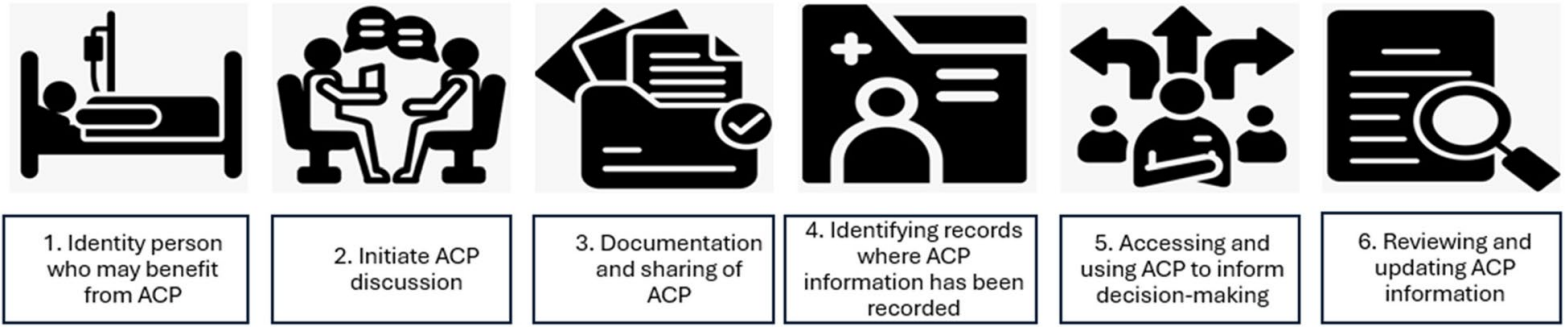
Different aspects of ACP

### Critical appraisal of individual sources of evidence

To assess the quality of evidence for each included study, the Grading of Recommendations, Assessment, Development, and Evaluations (GRADE) approach [24] was employed, which is a widely recognized framework for assessing the quality of evidence from research. However, GRADE ratings were not assigned to preprints due to their preliminary nature and lack of peer review, a standard process for journal articles. The GRADE ratings can be found in Appendix A.

### Synthesis of results

A narrative synthesis was conducted to outline the design, sample size, participant disease types, AI methods and primary and secondary aims of the included studies. The main features of ACP were then extracted and categorised into thematically similar groups. The performance of the AI approach was evaluated by two authors (UM, GM) and given a rating on a 5-point scale: Excellent (high accuracy and effectiveness in artificial intelligence approach), Very Good (suggesting solid model performance with some room for improvement), Good (demonstrating reasonable accuracy and effectiveness), Fair (moderate performance that may require further refinement), or Poor (significant room for improvement in the model's accuracy and effectiveness) (see Appendix C for further detail). The process of calculating performance levels for the models involved assessing each model’s performance based on several metrics, including area under the curve – receiver operating characteristic (AUC-ROC), F1 Score (a score of model accuracy), Accuracy, Precision and Recall, and Brier Score. The process of calculating performance levels for the models involves assessing each model’s performance based on several metrics, including AUCROC, F1 Score, Accuracy, Precision and Recall, and Brier Score. However, a key challenge arises when certain metrics are missing or unavailable, making it difficult to assign an accurate performance level based solely on incomplete data. To address this issue, we have developed a method where each individual metric is assessed separately for its performance level. This allows us to assign a level based on the available metrics. In cases where not all metrics are provided, we still assign a performance level for the ones available, ensuring that the evaluation is based on the best available data. Once individual performance levels for each metric are determined, a total performance level is calculated by considering the minimum value of the individual levels. This ensures that the final performance level reflects the model’s overall performance, even if some metrics are missing or unavailable.

This paper presents a novel approach to evaluating AI/ML models in scenarios where complete performance metric data is unavailable. Traditional methods often rely on a comprehensive set of metrics to assess model performance. However, when data is incomplete, accurate evaluation becomes challenging. Our proposed method addresses this limitation by evaluating each metric individually and assigning a performance level based on available data. The final performance level is determined by the minimum individual metric level, ensuring a conservative yet informative assessment of the model's overall performance. The data and source code availability were summarised. Finally, all studies were assessed for the clinical application of the AI approach to ACP and these findings were discussed in a narrative synthesis.

## Results

### Selection of sources of evidence

The database search yielded 394 studies, which were reduced to 385 unique records after duplicates were removed. Of these, 321 were excluded during abstract screening because they did not include AI approaches or were irrelevant to ACP. Subsequent screening of the full text led to the exclusion of 26 further studies for similar reasons (see Fig.Â 2), resulting in 38 studies being included in the scoping review. Alongside databases of published research, seven preprint servers were searched, yielding a further 500 studies. Of these, 25 were selected for full-text review, from which 3 studies were included. A total of 41 studies were including in the review (Fig.Â 2). A comprehensive list of included studies and extracted data is reported in Appendix B.

**Fig.Â 2:**
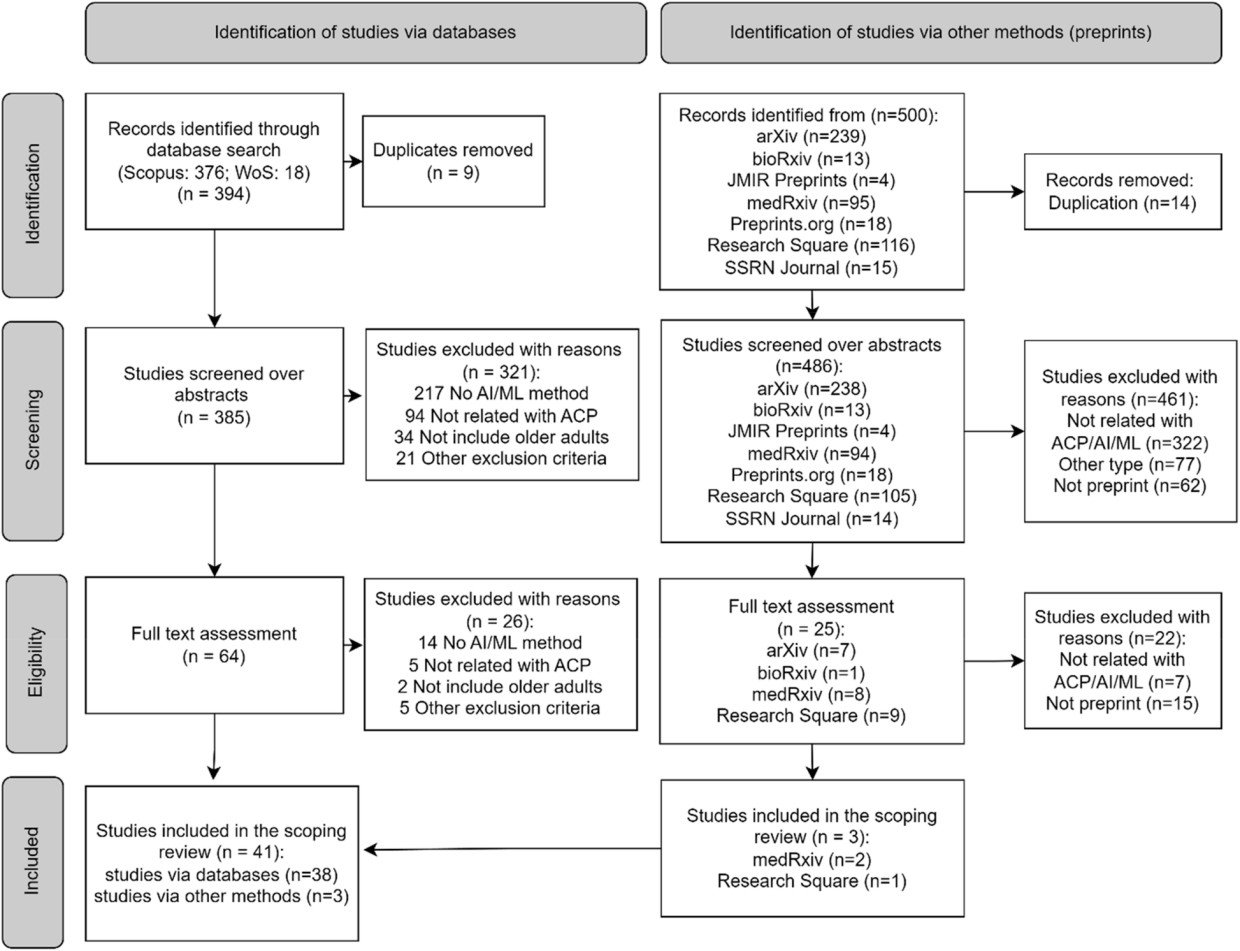
PRISMA flowchart of scoping review

### Critical appraisal within sources of evidence

According to the GRADE assessment, the majority of studies (*n* = 28; 68.42%) were rated as being of moderate quality. Two studies [12, 38] were rated as high quality and five studies [19, 25, 39–41] as low quality. In keeping with scoping review methodologies, these were not excluded.

### Results of individual sources of evidence

#### Study designs

Most studies (*n* = 24; 58.55%) utilized retrospective cohort designs (Table 1). Real-world EHR data was the most common data source (*n* = 37; 90.24%) for developing and training models, with varying sample sizes. The focus of studies varied, with'All Diseases'being the most frequent category (*n* = 24; 58.53%) (Table 1). Other common disease areas included cancer, Alzheimer's disease, and geriatric fragility fractures.

**Table 1:** Number of study design types used in included studies

Study design	*n*	Disease	n
Retrospective cohort study	24	All diseases	24
Prospective cohort study	6	Cancer	13
Mixed Methods Study	4	Alzheimer’s disease	2
Prospective observational study	3	End-stage liver disease	1
Randomized controlled trial	3	Geriatric fragility fracture	1
Cross-sectional study	1		

#### Characterising artificial intelligence approaches for ACP

The 41 studies included in this scoping review employed a diverse array of AI approaches to address aspects of ACP (see Appendix B). Within these, logistic regression (LR) [13–15, 20, 25, 39–46] and Random Forest models (RF) [12, 15, 19, 20, 25, 38, 40–42, 45–48] were the most widely used methods (see TableÂ 2). Artificial neural networks (ANN) [9, 14, 15, 40, 48–50], extreme gradient boosting (XGBoost) [13, 15, 39, 43, 51–53], support vector machines (SVM) [9, 13–15, 39, 44, 54], and decision trees (DT) [14, 15, 41, 43, 54–56] were also frequently employed. A range of other AI methods were also reported, including gradient boosting machines (GBM), deep neural networks (DNN), Gaussian naive Bayes (GNB), k-nearest neighbors (KNN), and natural language processing (NLP), where these methods were tailored to specific ACP contexts or tasks.

**Table 2:** Methods applied within AI approaches and aims of included studies

AI Method	Frequency (> 5)	Primary Aim	n	Secondary Aim	n
Logistic regression	13	Prediction	32	Decision Support	19
Random forest	13	Classification	4	Patient Selection	14
Artificial neural networks	7	Description	2	Knowledge Discovery	3
Decision trees	7	Optimization	2	Process Improvement	3
Support vector machines	7	Prediction and Classification	1	Optimization	2
Gradient boosting machines	7				
k-nearest neighbors	6				

The majority of studies (78%) detailed models that focused on forecasting or predicting future patient outcomes, such as survival, hospital length of stay, or the likelihood of specific events (TableÂ 2). These studies focussed on categorizing data into discrete classes or groups. Four studies [19, 48, 57, 58] involved classifying patients into high-risk or low-risk groups, predicting the presence or absence of a disease, or determining the optimal course of treatment. Studies in this category aimed to describe or summarize data without making specific predictions or classifications. This involved identifying patterns, trends, or relationships within the data. Two studies [21, 47] were categorized as descriptive, and focussed on finding the best possible solution to a given problem, such as optimizing treatment plans or resource allocation. One study [54] combined elements of both prediction and classification, aiming to both forecast outcomes and categorize patients.

The included studies were further classified according to their secondary aims (TableÂ 2). The most common secondary aim (46.34%) was decision support, alongside studies with a focus on selection of individuals who may benefit from specific interventions or treatments (41.17%). Additional secondary aims across studies included the discovery of new knowledge or insights from patient data (e.g., identifying patterns, trends, or relationships that were previously unknown; 7.31%) and optimising treatment plans or resource allocation (4.87%). A focus on improving processes within the healthcare system, such as streamlining workflows or optimising resource allocation, was also identified (7.31%).

#### Important features for ACP

Below, we outlined a comprehensive overview of the data elements utilised across AI models for ACP (detailed further in Appendix B). These features can be categorised into several key dimensions:

##### Patient-centric features

*Demographics and Socioeconomic Factors*: Age [9, 27, 29, 42, 43, 51], gender [13, 39, 51, 55, 56], and location [20, 39].

*Clinical Data*: A broad spectrum of clinical information, including diagnoses [10, 14, 53], procedures [10, 16], medications [10, 16, 57, 59], laboratory results [10, 41, 45, 52–54, 59], vital signs [10], medical history [16, 60], and symptoms (frailty [59], activity [22, 39, 43], pain [51, 57, 61], nausea [57], and delirium [13, 61]), was employed.

*Palliative and End-of-Life Indices*: Factors related to end-of-life care, including palliative care consultations [12], and do-not-resuscitate orders [62], largely used for predicting patient illness trajectories.

*Behavioral Data*: Studies incorporated patient activity data [22, 39, 43], sleep patterns [45], and body movements [19, 63].

##### Healthcare system features

*Resource Utilization*: Information about healthcare costs [14], service utilization (e.g., medical care, nursing) [50, 60], and system efficiency (e.g., length of stay, readmissions) [12] to optimize care delivery.

*Provider-Related Factors*: Information about healthcare providers, such as their experience [20], opinions [20], and work habits [38].

#### Model performance of the AI approaches

Within the studies that provided performance measure data, most of the studies (36.6%) demonstrated excellent performance. Some studies reported very good performance (30%) and good performance (16.6%). None of the studies were classified as fair, with some studies (*n* = 15; 36.6%) lacking sufficient information to assess their performance level. Particularly, traditional AI/ML models, like RF [9, 20, 43, 44, 55, 62], LR [9, 20, 40, 43, 44], SVM [9, 43, 44] and DT [43, 55] consistently demonstrated strong performance in the included studies, achieving ‘excellent’ results (level 1).

#### Data availability

The availability of data for the 41 studies varies significantly (see Appendix B). One study [64] made the source code publicly available, facilitating replication and further analysis. Some studies (*n *=15; 36.6%) indicated that data would be available upon reasonable request. However, many (*n *=17; 41.5%) did not provide any information regarding data availability. A small number of studies (*n* =5; 12.2%) explicitly stated that the data is not publicly available, potentially due to privacy or other concerns. Only one study provided an open dataset [16], making it accessible to other researchers for replication and further analysis. Three studies [57–59] Â provided a link to the dataset, facilitating access for interested parties. However, a significant majority of studies (*n *=32; 78.04%) did not provide any information regarding source code availability.

#### Clinical application of artificial intelligence approaches for ACP

The detailed classification of the different elements of ACP in the included studies is reported in Appendix D. Almost all studies focused on identifying individuals who might benefit from ACP (*n* = 39; 95.1%), while fewer studies addressed initiating ACP discussions (*n* = 10; 24.3%) or documenting and sharing ACP preferences and decisions (*n* = 8; 19.5%). Few studies explored where ACP discussions are recorded (*n* = 2; 4.87%). The next most frequently studied aspect was accessing and using ACP to inform decision-making (*n* = 37; 90.2%). No studies directly addressed reviewing and updating ACP information. The tools reported across the included studies primarily focused on supporting the identification of patients who might benefit from ACP and informing decision-making. No studies were identified where models support the reviewing and updating of ACP information (Fig. 3).

**Fig.Â 3:**
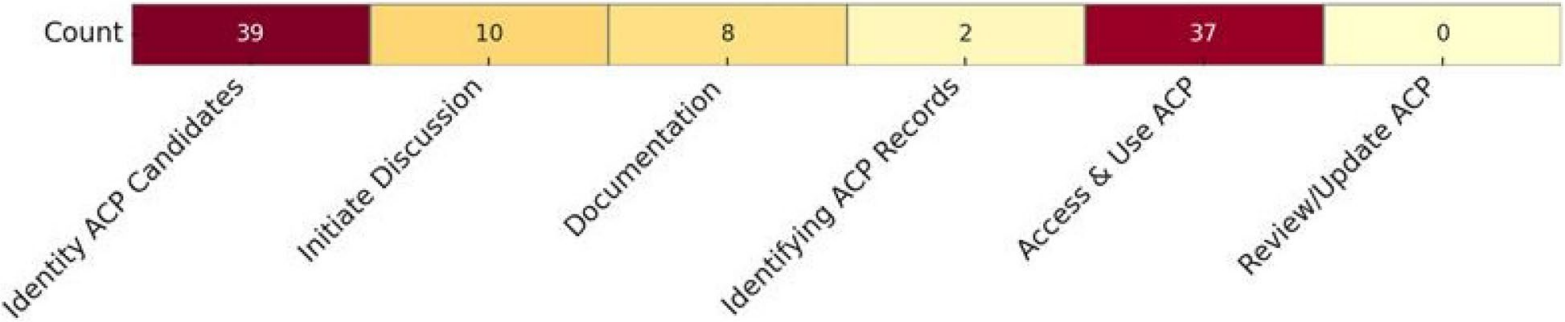
Heatmap for AI support across ACP phases

## Discussion

### Summary of evidence

This review provides an overview of the current literature detailing AI approaches for ACP and provides an overview of the current state, objectives, and critical features of these models. Overall, the scoping review demonstrates a strong focus on prediction and classification within the context of AI approaches in ACP. Included studies commonly focused on exploring how to embed AI approaches to assist healthcare professionals in making informed decisions about patient care and identifying patients who would benefit most from specific interventions or treatments. Wider secondary aims also highlighted AI approaches beyond prediction and classification, focusing on discovering new knowledge and improving healthcare processes. Most included studies were assessed as achieving an excellent performance, indicating the exceptional development of AI approaches for supporting ACP. However, while most studies performed very well, there is limited data availability and source code to ensure the reproducibility and transparency of emerging AI approaches for ACP research. As we will reinforce in the methods section, the findings of this review regarding AI approaches in ACP have applicability to broader contexts beyond palliative care, encompassing all individuals facing serious or life-limiting illnesses.

Most tools reported across the included studies are primarily focused on supporting the identification of patients who might benefit from ACP and informing decision-making, suggesting AI-based tools may be well-suited for identifying patients for whom discussion of end-of-life care preferences may be appropriate. This includes, for example, models to predict mortality and frailty for older patients to guide data-driven decision-making about the initiation of the ACP process. This may provide a prompt for earlier identification of opportunities to initiate ACP discussions and documentation, but there is recognition that such screening approaches may need to be developed to also anticipate palliative care needs and predict the rate and course of functional decline [65]. There was, however, gaps in the coverage of AI approaches to support different phases of ACP, including during ACP discussions and ACP documentation and sharing. In busy clinical practice, clinicians may struggle to find time to support ACP at the patient’s preferred pace. A 2019 systematic review described patient-level barriers to ACP, such as perceived irrelevance and poor awareness, and also time issues for clinicians [66]. Approaches such as using AI-powered chatbots are being considered for offering resources for spiritual exploration and facilitating religious practices for people living with head and neck cancer [67]. In the context of ACP, similar AI-powered chatbots may have potential utility for supporting exploration and documentation of ACP preferences, assisting patients across different elements of ACP, which could potentially reduce clinical workload while empowering patients. The review highlighted a notable gap in literature detailing the role of AI-based approaches to support the reviewing and updating of ACP documentation. AI models have the ability to pick up clinical changes in real time [61, 68] and may have the potential to facilitate timely review within this aspect of ACP. For example, patient-directed chatbots or conversational agents could promote reflection and revision between clinical visits [12, 67]. The use of scenario modelling tools can aid patients to anticipate future care needs and adjust preferences where necessary [69]. Further, AI added to workflows can stimulate clinicians to re-initiate ACP conversations after key health events [42, 70]. Addressing this gap in AI-based ACP research should be a priority for future research.

An empowered patient who has accessed and processed a certain level of information about their disease and its likely future progression, and who has already reflected on their values, wishes and fears for the future, would likely derive more benefit from a time-limited interaction with a clinician than one who is uninformed and unprepared. AI approaches could support targeted, interactive information provision and future scenario modelling to the patient, supporting the patient to document their questions, decisions and uncertainties in advance of an ACP discussion with a clinician. A patient-driven approach also circumvents the known difficulties that clinicians report in finding ‘the right time’ to have ACP conversations [71], as the patient can interact with information relevant to them, in their own time, and be empowered to seek the ACP interaction, rather than waiting for a clinician to initiate it. AI could also enable real-time monitoring of patients’ health conditions and provide alerts to consider adjustments to ACP documentation. This use of AI could embed ACP as a continual process, rather than a one-time event [72].

There are points that should be considered when implementing AI approaches in a healthcare system. While AI approaches could offer data-driven insights that may assist clinicians in making informed decisions and improve the quality of ACP discussions, they should augment clinical skills rather than supplant clinical decision-making [73]. Furthermore, patients’ end-of-life care choices can be shaped by cultural factors [74], therefore, AI systems should be intentionally designed to accommodate a wide range of cultural backgrounds, as there may be variability when it comes to how and who should be involved in ACP discussions. For example, the development of AI-based approaches could utilise diverse datasets, ACP content may be personalised in response to known or disclosed cultural or religious affiliation (e.g., for a Muslim patient presenting content addressing fasting during illness), prioritise the ability of systems to interpret and respond when people use religious and culturally nuanced terms, and ensuring the co-design and development of AI-based approaches with input and involvement of patient and communities from diverse backgrounds. There is also scope to draw on emerging frameworks such as a focus on adopting AI approaches in culturally diverse healthcare settings to avoid culturally inappropriate discussions [75]. The use of AI in healthcare raises ethical concerns, such as data privacy, informed consent, and algorithmic bias. It is essential that AI systems are transparent and that patients’ data are safeguarded to ensure patients can have continued trust in their healthcare [76].

The findings of this scoping review highlight the potential of AI approaches to support ACP. However, despite the model performance of some of the artificial intelligence approaches being categorised as good or excellent, this does not hold across all the models. Limitations in the performance of the models can be attributed to the quantity and diversity of the training sample available for the AI/ML models to use. This scoping review identified only one study providing an open dataset. Promoting open-source practices is essential for improving transparency, collaboration, and the overall quality of ACP research with AI-based approaches [77] and is an increasing requirement of research funders. While several studies acknowledge the use of proprietary datasets or models without sharing source code, this limited transparency poses significant challenges. Specifically, it hampers reproducibility, as other researchers cannot verify or replicate the reported findings. Furthermore, the inability to inspect training data raises concerns about potential bias, which may undermine the fairness and generalizability of AI systems. Finally, without open access to code or validation data, independent assessment of model performance is not possible, limiting the credibility of these approaches in clinical settings. Furthermore, it is suggested that a range of features used within these models can help improve their performance including patient-level variables (functional status and symptoms) [78, 79], healthcare utilisation variables (resource utilisation, patient satisfaction) and healthcare professional factors (experience and opinions) [13]. Therefore, the diversity of the sample and inputs is key to improving model performance.

This review identified that most studies focussed on all disease types when building the models for AI use in ACP. While this approach can make the models generalisable to multiple patient populations, it also requires the model to be more complex as it requires an understanding of disease-specific factors and variations which can be a challenge to finding samples of sufficient size and quality to train the models. Future research focusing on disease-specific models of ACP may help in developing more effective and tailored tools to support decision-making that addresses the specific needs of different disease populations. For example, research has highlighted the different barriers in ACP for certain disease populations, including a lack of understanding of the disease trajectory by the healthcare professional, treatment option availability, and reduced mental capacity in the patient [80]. The latter is particularly important in dementia, a common disease where ACP is needed as the person will lose communication and decision-making abilities as the disease progresses [81]. AI approaches could also support ACP as a more continuous process, as is known best practice [82], rather than a discrete, rushed event. AI approaches could be particularly beneficial in atypical dementia sub-types, where a person may be showing signs of impaired speech, understanding or concentration early in the disease, and AI could flag up the need for more urgent ACP conversations [82], supporting simple information being presented in the means that best suited the person’s information processing needs at the time. Therefore, by accurately predicting future outcomes and classifying patients into appropriate categories, AI may help healthcare providers initiate timely ACP, alongside providing tailored information to guide ACP discussions and documentation.

Our review identified a diverse range of applications of AI models in the context of ACP. To provide a more nuanced understanding of these applications, we categorized the included studies based on the directness of their support for ACP: Category 1) Direct ACP Support: AI models designed to directly facilitate or perform core ACP activities (e.g., eliciting preferences, supporting shared decision-making, generating ACP documents). Category 2) Indirect ACP Support: AI models that provide information or perform tasks that support ACP but do not directly carry out the core activities (e.g., predicting events that might trigger ACP discussions, identifying patients who might benefit from ACP) and Category 3) Tangential Relevance: AI models with a more tangential or distant relationship to ACP, focusing on related areas (e.g., palliative care more broadly) but potentially having implications for ACP.

Our analysis suggests that many included studies fall into Categories 2 and 3. For example, Study #4 used AI to predict patient mortality as a trigger for ACP discussions, while other studies focused on predicting patient outcomes or managing symptoms in palliative care settings. While these applications can contribute to the context of ACP, they do not always directly engage with the core elements of ACP itself. The limited number of studies that explicitly focused on Category 1 (direct ACP support) highlights a potential gap in the current literature.

This observation has several implications: * The current evidence base may be stronger for AI's ability to support ACP-related tasks than for its ability to directly facilitate core ACP processes. * The potential of AI to transform ACP by directly engaging with patients in preference elicitation or shared decision-making remains largely unexplored. * Future research should prioritize the development and rigorous evaluation of AI models that actively support the core elements of ACP.

### Limitations

While the search strategy was comprehensive, there are certain limitations. The search was conducted in Scopus and Web of Science databases, covering a large proportion of existing health research literature. However, the omission of CINAHL may have resulted in the omission of potentially relevant articles outlining clinical implementation and nursing perspectives on AI-based relating to CP. Limiting the search to English, German, and French may have excluded studies published in other languages. This review focused on published work in academic journals and preprints, which may have excluded relevant studies in the grey literature. During data extraction, researchers may inadvertently introduce bias during the process due to factors such as preconceived notions, selective interpretation of data, or variations in how data is coded. To minimize this, we employed a standardized data extraction form, cross-checked extracted data, and ensured inter-rater reliability.

## Conclusion

AI-based approaches are being developed for ACP, with the majority focusing on predictions to guide the timely initiation of ACP discussions. Beyond prediction and classification, AI models are also being explored for decision support, but few studies explore supporting the initiation of ACP discussions, or processes around the documentation and sharing of ACP information. There is a gap in the evidence base for the role of AI-based approaches in supporting the reviewing and updating of ACP information, either undertaken with health professionals, or through patient-facing resources that may be completed alone or alongside caregivers. To address these gaps and advance the field, future research should prioritize several key areas: encourage the creation and sharing of open-access AI datasets specifically for ACP. This would enhance the transparency of AI models, facilitate reproducibility, and enable more rigorous scrutiny and validation of their performance. It is also important to explore the development of AI-driven ACP revision systems that can facilitate real-time patient engagement. These systems could leverage AI to dynamically update ACP documents based on changes in patient health status, preferences, or values, ensuring that ACP remains a living document. Furthermore, there is a need to create AI tools that support culturally sensitive ACP discussions. This includes incorporating natural language processing (NLP) to understand and respond to diverse communication styles, values, and beliefs related to end-of-life care, promoting more inclusive and equitable ACP practices. Further research is also needed to investigate AI applications that can effectively support the initiation of ACP discussions. This could involve AI tools that can identify optimal timing for these conversations, assist clinicians in framing ACP discussions, or provide patients with tailored information and resources to prepare for ACP. Alongside exploring novel AI approaches within ACP, there is a need to consider the underpinning transparency and quality of emerging AI-based approaches for ACP, with a mixed performance of the reported models, and a lack of data and source code that would facilitate reproducibility and scrutiny. By focusing on these recommendations, the field can move towards developing more robust, equitable, and impactful AI applications to enhance advance care planning.

## Data Availability

No datasets were generated or analysed during the current study.

## Appendix 1

**Table:**

Study No	Study	GRADE Rating	Study No	Study	GRADE Rating	Study No	Study	GRADE Rating
1	[17]	Moderate	14	[43]	Low-to-Moderate	27	[40]	Low
2	[39]	Low	15	[47]	Moderate	28	[51]	Moderate
3	[56]	Moderate	16	[44]	Moderate	29	[45]	Low-to-Moderate
4	[10]	Moderate	17	[16]	Moderate	30	[19]	Low
5	[49]	Moderate	18	[48]	Low-to-Moderate	31	[59]	Moderate
6	[50]	Moderate to high	19	[60]	Moderate	32	[41]	Low
7	[15]	Moderate	20	[69]	Moderate	33	[58]	Moderate
8	[25]	Low	21	[54]	Moderate	34	[55]	Moderate
9	[83]	Moderate	22	[38]	High	35	[52]	Moderate
10	[22]	Moderate	23	[57]	Moderate	36	[9]	Moderate to high
11	[63]	Moderate	24	[64]	Moderate	37	[21]	Moderate
12	[61]	Moderate	25	[14]	Moderate	38	[62]	Moderate
13	[12]	High	26	[20]	Moderate			

## Appendix 2

**Table:**

**No**	**Study**	**Year**	**Disease**	**Study Design**	**Number of participants**	**Dataset source**	**Number of Records**	**AI/ML Models**	**Primary Aim**	**Secondary Aim**	**Data Types Included in Models**	**Model Performance**	**Data Availability**	**Source Code**
1	[17]	2021	All Diseases	Prospective cohort study	178	real-world EHR	97,683	Gboost	Prediction	Patient Selection	Eligibility issues, limited ACP resources, early patient discharge, lack of outpatient follow-up, and comparison of outpatient vs. inpatient ACP effectiveness	AUCROC: 0.86, AUPRC: 0.76	Not publicly available	No info
2	[39]	2021	Alzheimer’s disease	Retrospective cohort study	-	Facebook	243	LR, GBoost, SVM, RF, KNN, XGBoost, GNB	Prediction	Optimization	Gender, age at diagnosis, message frequency, behavior, activity, caregiver, location, and relationship	GNB F1 score: 0.85, SVM F1 score: models: 0.79, LR F1 score: 0.69	No info	No info
3	[56]	2024	Cancer	Mixed Methods Study	640	No info	No info	DT	Prediction	Decision Support	Age, sex, cancer type, and treatment type	No info	Available upon reasonable request	Available upon reasonable request
4	[10]	2023	All Diseases	Mixed Methods Study	201	real-world EHR	18,631	DL	Prediction	Patient Selection	Patient demographics, diagnoses, procedures, medications, lab results, vital signs, and social background	AUCROC: 0.89	Not publicly available	No info
5	[49]	2024	Geriatric fragility fracture	Retrospective cohort study	-	real-world EHR	7,605	GBoost, ANN, DL	Prediction	Patient Selection	Patient background, medical evaluation, hospital admission details, surgery information	Model 1: GBoost AUCROC: 0.73, F1 score: 0.68; Model 2: ANN accuracy: 0.76, F1 score: 0.64; Model 3: DL accuracy: 0.79, precision: 0.73, AUCROC: 0.84	No info	No info
6	[50]	2020	All Diseases	Mixed Methods Study	No info	personal photos	No info	ANN	Description	Process Improvement	Patient position in bed and nurse actions	No info	No info	No info
7	[15]	2021	All Diseases	Retrospective cohort study	-	real-world EHR	1,462,862	SVM, ANN, LR, RF, DT, XGBoost	Prediction	Patient Selection	Patient characteristics	(Best Model) LR accuracy: 0.64, 0.68, 0.60 for all-cause cohort, positive and negative classes	No info	No info
8	[25]	2024	All Diseases	Retrospective cohort study	-	real-world EHR	No info	LR	Prediction	Patient Selection	No information provided	No info	No info	No info
9	[83]	2022	All Diseases	Retrospective cohort study	-	real-world EHR	19,753	GBM, DNN	Prediction	Decision Support	Patient survival and frailty over one year	1-year mortality classifier AUCROC: 0.87, 1-year frailty classifier AUC ROC: 0.89	Provides link	Provides link
10	[22]	2023	Cancer	Prospective observational study	78	real-world EHR	66	Transformer, LSTM, BiLSTM, GRU	Prediction	Decision Support	Patient activity data and medical information	Accuracy: 0.878 and 0.924 for 12 and 24 hperiod	Available upon reasonable request	No info
11	[63]	2021	Cancer	Prospective cohort study	60	real-world EHR	44	LSTM	Prediction	Decision Support	Body movement measurements	Accuracy: 0.83	Available upon reasonable request	No info
12	[61]	2019	Alzheimer disease and related dementias (ADRD)	Retrospective cohort study	-	real-world EHR	26,921	LSTM	Prediction	Patient Selection	mental function, delirium, cholesterol testing, pain, healthcare use, nutrition, skin condition, family support, severe medical problems, and swallowing difficulties	The 6-month model AUROC: 0.978; the 1-year model AUROC: 0.956; the 2-year model AUROC: 0.943	No info	No info
13	[12]	2023	All Diseases	Randomized controlled trial	3183	real-world EHR	755	Predictive modeling	Prediction	Patient Selection	Palliative care consultation note, hospital readmissions, length of stay	Reduced 60-day and 90-day hospital readmissions: Odds Ratio: 0.75 and 0.72, respectively	Available upon reasonable request	No info
14	[43]	2023	Cancer	Prospective cohort study	40	real-world EHR	40	LR, SVM, DT, RF, KNN, AdaBoost, XGBoost	Prediction	Patient Selection	Heart rate, physical activity, eating, urination, and stage of illness	(best model) XGBoost AUROC: 96%, F1-score: 78.5%, accuracy: 93%, and specificity: 97%	Available upon reasonable request	No info
15	[47]	2021	All Diseases	Retrospective cohort study	-	real-world EHR	70,788	RF based 4 models	Optimization	Process Improvement	Improvement assessment, patient usage and net benefit	(RF-1, RF-2, RF-3, RF-4) Sensitivity: 0.37, 0.28, 0.29, 0.26 Specificity: 0.95, 0.94, 0.94, 0.93 Brier: 0.11, 0.12, 0.12, 0.12	Not publicly available	Available upon reasonable request
16	[44]	2021	All Diseases	Retrospective cohort study	-	real-world EHR	120,940	LR, RF, SVM	Prediction	Patient Selection	Patient age, overall health, and how the patient arrived at the hospital	(unsurprising deaths) LR, RF, and SVM AUROC: 0.95, 0.94, 0.94. (All mortality) LR, RF, and SVM AUROC: 0.92, 0.92, 0.93	No info	No info
17	[16]	2018	All Diseases	Retrospective cohort study	-	STRIDE	221,284	DNN	Prediction	Patient Selection	Patient medical history, procedures, medications, and hospital visits	Precision: 0.69, recall: 0.34, AUROC: 0.93	Open dataset	No info
18	[48]	2022	All Diseases	Mixed Methods Study	751	real-world EHR	751	ANN, GNB, DF, ID3, LMT, RF	Classification	Decision Support	Patient symptoms and overall condition	(Stable, Unstable, Deteriorating and Terminal) AUCROC: 0.639, 0.60, 0.627, 0.724	Available upon reasonable request	No info
19	[60]	2022	Cancer	Retrospective cohort study	-	real-world EHR	743	LSTM	Prediction	Optimization	medical care, nursing, psychology, rehabilitation, spiritual support, social work, personal history, and medical equipment	accuracy: 69.75% and F1 score: 66.8%	No info	No info
20	[69]	2024	Cancer	Retrospective cohort study	-	real-world EHR	2314	XGBoost, Gboost, AdaBoost, LR, SVM, RF	Prediction	Knowledge Discovery	Gender, previous delirium, cancer treatment, smoking, alcohol use, and living situation	(best model) XGBoost + RF sensitivity: 0. 68, specificity: 0.70, balanced accuracy: 0.69, AUCROC: 0.74	Available upon reasonable request	Provides link
21	[54]	2020	End-stage liver disease (ESLD)	Retrospective cohort study	-	real-world EHR	1903	LDA, SVM, GNB, DT, RF, AdaBoost	Prediction and Classification	Decision Support	Blood test results related to kidney, liver, and fluid balance	(best model) RF AUCROC: 0.852, AdaBoost AUCROC: 0.833	No info	No info
22	[38]	2022	Cancer	Randomized controlled trial	2695	real-world EHR	26,059	GBoost	Prediction	Patient Selection	Personal information, work habits, and end-of-life care practices	model performance: 11.6% increase	No info	No info
23	[57]	2021	All Diseases	Retrospective cohort study	-	HOPE	9924	ARM	Classification	Decision Support	Pain, nausea, and medications	model performance: 23.6% increase, AUCROC: 0.89	Provides link	No info
24	[64]	2019	All Diseases	Retrospective cohort study	-	real-world EHR	33,509	LSTM	Prediction	Decision Support	Patient medical codes and doctor notes	performance increase versus baseline model, the frequency model: 6%; entropy model: 5%; word2vec model: 15%. (best model versus doctors') keyword model: 9% increased accuracy	Available upon reasonable request	Publicly available
25	[14]	2021	All Diseases	Retrospective cohort study	-	real-world EHR	17,197	DT, RF, SVM, LR, ANN, AdaBoost	Prediction	Decision Support	Patient diagnoses, healthcare costs and patient information	(best model) AdaBoost precision: 0.71, recall: 0.67	No info	No info
26	[20]	2022	All Diseases	Cross-sectional study	3505	real-world EHR	3505	LR, RF	Description	Knowledge Discovery	Opinions, work environment, confidence, job title, hospice experience, work location, and doctor occupation	RF accuracy: 0.75, F1 score: 0.84, recall = 0.94	Available upon reasonable request	No info
27	[40]	2022	Cancer (lung cancer)	Prospective cohort study	80	real-world EHR	400	LR, ANN	Prediction	Decision Support	Bone marrow problems, hospital stay, age, diabetes, chemotherapy, surgery, and hormone treatment	LR AUCROC: 0,729, ANN AUCROC: 0,897	Available upon reasonable request	No info
28	[51]	2024	Cancer	Retrospective cohort study	-	real-world EHR	561	XGBoost	Prediction	Decision Support	Pain level, calcium level, age, sex, and falls	AUCROC: 0.89, sensitivity: 95.8, specificity: 0.71	No info	Available upon reasonable request
29	[45]	2023	Cancer	Prospective observational study	49	real-world EHR	49	LR	Prediction	Decision Support	Sleep patterns, doctor's opinion, overall health, and blood test results	The predicted median hazard: 0.00052, Pearson’s corr. Coef rr = − 0.08; p = 0.5808	Available upon reasonable request	No info
30	[19]	2023	All Diseases	Prospective observational study	14	real-world EHR	14	CNN, LSTM	Classification	Decision Support	Body movements while doing everyday tasks	Accuracy: 99.8	No info	No info
31	[59]	2021	All Diseases	Retrospective cohort study	-	real-world EHR	19,753	RF	Prediction	Decision Support	Age, medications, overall health, function, kidney function, blood cell count, and cancer spread	AUCROC: 0.83	No info	Provides link
32	[41]	2023	All Diseases	Retrospective cohort study	-	real-world EHR	113	LR, DT	Prediction	Decision Support	Blood test results	Model significance: p = 0.001 (adjusted R2 = 0.15)	Available upon reasonable request	No info
33	[58]	2022	All Diseases	Retrospective cohort study	-	real-world EHR	104	CFA	Classification	Decision Support	Patient's feelings about treatment	patients’ overall state change: higher than control group. The average satisfaction: 20% higher than control group	Provides link	No info
34	[55]	2018	Cancer (colon or rectal cancer)	Retrospective cohort study	-	SEER	27,795	KNN, GNB, DT, RF	Prediction	Decision Support	Age, tumor size, cancer stage, number of lymph nodes, surgery, and gender for rectal cancer	(1-Year 2-Year 3-Year 4-Year 5-Year Average) colon cancer (AUROC: 0.980; 0.984; 0.986; 0.988; 0.985; 0.9846) rectal cancer (AUROC: 0.957; 0.960; 0.961; 0.963; 0.971; 0.9608)	No info	No info
35	[52]	2024	Cancer	Prospective cohort study	5926	real-world EHR	52,538	XGBoost	Prediction	Decision Support	Patient information, medical information, lab results, cancer treatment, and healthcare use	AUROC: 0.861, AUPRC: 0.771, The Brier score: 0.147	Available upon reasonable request	Provides link
36	[9]	2019	All Diseases	Retrospective cohort study	-	real-world EHR	120,940	LR, SVM, KNN, RF, ANN, Gboost	Prediction	Patient Selection	Patient age, overall health, and how the patient arrived at the hospital	AUCROC: 0.95, sensitivity: 0.87, specificity: 0.86	Not publicly available	Available upon reasonable request
37	[21]	2023	All Diseases	Prospective cohort study	24	No info	No info	CDSS	Optimization	Process Improvement	How easy the system is to use and user experience	SUS: 62.7 ± 14.1 and 65 ± 26.2. UEQ-S: 1.42 and 1.5	Available upon reasonable request	No info
38	[62]	2020	All Diseases	Retrospective cohort study	-	real-world EHR	36,368	RF	Prediction	Knowledge Discovery	Reasons for hospital visits, including palliative care, do-not-resuscitate orders, and other care	AUCROC: 0.9477	No info	No info
39	[53]	2023	Cancer	Retrospective cohort study	-	real-world EHR	38,494	XGBoost	Prediction	Decision Support	Demographics, 6-month time series of lab test results and flowsheet data, and diagnoses	AUROC: 0.83, AUPRC: 0.4, The Brier score: 0.08	No info	No info
40	[46]	2021	All Diseases	Randomized controlled trial	-	real-world EHR	-	Bayesian estimation, LR	Prediction	Patient Selection	Patient EMR information	No info	Will be available upon reasonable request	No info
41	[18]	2021	All Diseases	Retrospective cohort study		real-world EHR	Approx. 5Â M	NLP	Prediction	Patient Selection	Patient documents	No info	Not publicly available	No info

## Appendix 3

### Performance Levels of AI Models in The Included Studies

**Table:**

Study No	Study	Performance Level	Study No	Study	Performance Level	Study No	Study	Performance Level
1	[17]	Level 2	15	[47]	Level 4	29	[45]	Level 2
2	[39]	Level 2	16	[44]	Level 1	30	[19]	No information
3	[56]	No information	17	[16]	Level 1	31	[59]	Level 1
4	[10]	No information	18	[48]	Level 3	32	[41]	Level 3
5	[49]	Level 3	19	[60]	Level 4	33	[58]	No information
6	[50]	No information	20	[69]	Level 4	34	[55]	No information
7	[15]	Level 4	21	[54]	Level 2	35	[52]	Level 1
8	[25]	No information	22	[38]	No information	36	[9]	Level 2
9	[83]	Level 2	23	[57]	Level 2	37	[21]	Level 1
10	[22]	Level 2	24	[64]	Level 4	38	[62]	Level 2
11	[63]	Level 3	25	[14]	Level 3	39	[53]	Level 1
12	[61]	Level 1	26	[20]	Level 1	40	[46]	Level 1
13	[12]	No information	27	[40]	Level 1	41	[18]	No information
14	[43]	Level 1	28	[51]	Level 4			

Given the diversity of metrics and the potential for varying interpretations, it's challenging to provide a definitive 5-level classification. However, we can offer a general assessment based on common performance thresholds based on literature [84, 85]:

**Table:**

**Level 1 (Excellent):** • AUCROC > 0.9 • F1 Score > 0.9 • Accuracy > 0.9 • Precision and Recall > 0.9 • Brier Score < 0.1	**Level 2 (Very Good):** • AUCROC between 0.85 and 0.9 • F1 Score between 0.8 and 0.9 • Accuracy between 0.8 and 0.9 • Precision and Recall between 0.8 and 0.9 • Brier Score between 0.1 and 0.15	**Level 3 (Good):** • AUCROC between 0.8 and 0.85 • F1 Score between 0.7 and 0.8 • Accuracy between 0.7 and 0.8 • Precision and Recall between 0.7 and 0.8 • Brier Score between 0.15 and 0.2
**Level 4 (Fair):** • AUCROC between 0.7 and 0.8 • F1 Score between 0.6 and 0.7 • Accuracy between 0.6 and 0.7 • Precision and Recall between 0.6 and 0.7 • Brier Score between 0.2 and 0.25	**Level 5 (Poor):** • AUCROC < 0.7 • F1 Score < 0.6 • Accuracy < 0.6 • Precision and Recall < 0.6 • Brier Score > 0.25	

## Appendix 4

**Table:**

Study No	Study	Directness of their support for ACP	Identity person who may benefit from ACP	Initiate ACP discussion	Documentation and sharing of ACP	Identifying records where ACP information has been recorded	Accessing and using ACP to inform decision-making	Reviewing and updating ACP information
1	[17]	2	X	X			X	
2	[39]	3	X				X	
3	[56]	3	X		X			
4	[10]	2		X	X	X	X	
5	[49]	2	X				X	
6	[50]	3	X				X	
7	[15]	2	X				X	
8	[25]	2	X				X	
9	[83]	3	X				X	
10	[22]	3	X				X	
11	[63]	3	X				X	
12	[61]	2	X				X	
13	[12]	2	X	X	X		X	
14	[43]	2	X	X	X		X	
15	[47]	2	X	X	X		X	
16	[44]	2	X				X	
17	[16]	2	X				X	
18	[48]	3	X				X	
19	[60]	3	X		X		X	
20	[69]	3	X				X	
21	[54]	2	X				X	
22	[38]	2	X	X			X	
23	[57]	3	X				X	
24	[64]	2	X	X			X	
25	[14]	2	X		X		X	
26	[20]	3		X				
27	[40]	3	X				X	
28	[51]	2	X				X	
29	[45]	2	X				X	
30	[19]	3	X				X	
31	[59]	2	X				X	
32	[41]	3	X				X	
33	[58]	3	X				X	
34	[55]	2	X				X	
35	[52]	2	X				X	
36	[9]	2	X				X	
37	[21]	3	X				X	
38	[62]	3	X				X	
39	[53]	2	X	X				
40	[46]	2	X					
41	[18]	1	X	X	X	X	X	
			**39**	**10**	**8**	**2**	**37**	**0**

## Abbreviations

ACP: Advance Care Planning
AdaBoost: Adaptive boosting
AI: Artificial Intelligence
ANN: Artificial Neural Networks
ARM: Association rule mining
AUPRC: Area Under the Precision-Recall Curve
AUROC: Area Under the Receiver Operating Characteristic Curve
BiLSTM: Bidirectional Long short-term memory
CFA: Collaborative filtering algorithm
CNN: Convolutional neural network
DF: Decision forest
DL: Deep Learning
DNN: Deep Neural Networks
DT: Decision Trees
EHR: Electronic Health Record
ESLD: End-stage liver disease
GBM: Gradient Boosting Machines
Gboost: Gradient Boost classifier
GNB: Gaussian NaÃ¯ve Bayes
GRU: Gated Recurrent Units
ID3: Iterative Dichotomiser 3
KNN: K-Nearest Neighbour
LDA: Linear discriminant analysis
LMT: Logistic model tree
LR: Logistic Regression
LSTM: Long short-term memory
ML: Machine Learning
NLP: Natural Language Processing
RF: Random Forest
SUS: System Usability Scale
SVM: Support Vector Machine
UEQ-S: User Experience Questionnaire – Short Version
XGBoost: Extreme gradient boosting
